# Auricular Point Acupressure for Chronic Low Back Pain: A Feasibility Study for 1-Week Treatment

**DOI:** 10.1155/2012/383257

**Published:** 2012-07-01

**Authors:** Chao-Hsing Yeh, Lung-Chang Chien, Yi-Chien Chiang, Li-Chun Huang

**Affiliations:** ^1^School of Nursing, University of Pittsburgh, 3500 Victoria Street, 440 Victoria Building, Pittsburgh, PA 15261, USA; ^2^Department of Internal Medicine, Washington University in St. Louis, St. Louis, MO, USA; ^3^Department of Nursing, Chang Gung Institute of Technology, Taiwan; ^4^World Academy of Auricular Medicine, FL, USA

## Abstract

*Objectives*. The objective of this one-group, repeated-measures design was to explore the acceptance of auricular point acupressure (APA) to reduce chronic low back pain (CLBP) and estimate minimum clinically important differences (MCIDs) for pain intensity change. *Methods*. Subjects received 7-day APA treatment. After appropriate acupoints were identified, vaccaria seeds were carefully taped onto each selected auricular point for 7-day. The Brief Pain Inventory Short Form (BPI) was used to collect outcome data. *Results*. A total of 74 subjects participated in the study. Ten subjects dropped out and the retention rate was 87%. Subjects reported a 46% reduction in BPI worst pain, and over 50% reduction in BPI average pain, overall pain severity and pain interference by the end of study, and 62.5% subjects also reported less pain medication use. The MCIDs for the subscale of BPI ranged from .70 to 1.86 points. The percentage improvement of MCIDs from baseline was between 14.5–24.9%. *Discussion*. APA appears to be highly acceptable to patients with CLBP. A sham group is needed in order to differentiate the true effects of APA from the possible psychological effects of more frequent visits by the auricular therapist and patients' expectation of the APA treatment.

## 1. Introduction

Chronic low back pain (CLBP) imposes a significant societal and economic burden on the United States. The prevalence of CLBP has increased from 3.9% in 1992 to 10.2% of the population in the United States in 2006 [1], with an estimated annual cost of $84.1 billion for direct treatment and an additional $624.8 billion as a result of loss of productivity [2, 3]. Despite the availability of more than 200 treatment options for CLBP [4], improvements in patient outcome are limited [5]. Acupuncture offers another treatment option for CLBP and has shown promising effects, namely, short-term pain relief [6, 7]. However, conflicts exist about the effectiveness between acupuncture alone and conventional therapies [8]. The widespread application of acupuncture to manage CLBP is limited by the need for patients to travel to the acupuncture site [9], fear of needles, and the cost of acupuncture treatment not typically being covered by insurance [10].

Auricular therapy, an adjunct to acupuncture, is based on the same ancient Traditional Chinese Medicine (TCM) as acupuncture and uses acupoints on specific areas of the inner and outer ear lobe to treat disease/illness [11, 12]. In TCM, a disease is considered to be caused by the imbalance of a person's energy, Qi [11]. The stimulation of auricular acupoints regulates Qi, activates the meridians and collateral systems, and has been successful in treating health problems [11, 12]. In the 1950s, a French neurosurgeon, Dr. Paul Nogier, theorized that the ear represents the inverted fetus within the womb, and proposed the somatotopic correspondence of specific parts of the body to specific parts of the ear [11, 12]; the current auricular therapy practiced worldwide is based on Nogier's theory. The World Health Organization considers auricular medicine a form of microacupuncture that can affect the whole body [13]. Although popular in Asia for over 2000 years and in Europe for the past 60, auricular therapy has not yet been widely practiced by health care providers in the United States. 

The most common auricular therapies include acupuncture, electroacupuncture stimulation, and acupressure. Studies using auricular therapy (acupuncture or acupressure on the external ear) have shown promising effects in pain management. A recent meta-analysis of auricular therapy for pain management (17 studies: 8 perioperative, 4 acute, and 5 chronic pain), found auricular therapy reduced analgesic use for perioperative pain (standard mean difference (SMD) = 0.54 [95% confidence interval (CI) 0.30, 0.77]), and reduced pain intensity for acute and chronic pain (SMD = 1.56 [95% (CI): 0.85, 2.26]) than for controls [14]; three of the studies were conducted in the USA [15–17]. Electrical stimulation of auricular points treatment has been found to be more effective than manual stimulation [18, 19]. When the auricular acupuncture was combined with exercise, the benefits for CLBP were increased even more [20, 21]. The needle of auricular acupuncture can stay in situ for up to 1 week and therefore reduce the number of therapist office visits. While these findings support the growing enthusiasm for this complementary and alternative approach to pain management, the authors note several limitations, including limited evidence from rigorous clinical trials, small sample sizes, and inadequate blinding procedures [22]. In addition, the practice of auricular acupuncture needs to be performed by licensed acupuncturists. 

Auricular Point Acupressure (APA) [11, 12] is a less frequently employed yet validated method to deliver auricular therapy, which utilizes very tiny botanical plant seeds (e.g., approximately 2 mm size) taped onto the patient's ear for acupoint stimulation. Once applied by a qualified therapist, the taped seeds remain in place for up to 1–3 weeks, depending on the subject's skin condition. The patient is instructed to apply pressure to the taped seed when experiencing pain. In addition, the selection of auricular acupoints for the APA treatment is individualized according to the corresponding body part exhibiting symptoms. In general, acupressure is less effective at the same duration, but acupressure can obtain the same benefits as acupuncture when it is applied for a longer time [23, 24]. APA is also adaptable to health care professional practice because it can be taught along the continuum of health professional education, which would enable more health care professionals to incorporate it into practice to provide pain relief and augment the effects of pain medication. 

In order to interpret the APA effects of pain intensity change (patient-reported measure) appropriately, minimum clinical important differences (MCIDs) have been suggested [25]. MCIDs represent the smallest change considered by the patient as an improvement. MCIDs may be estimated using anchor-based or distribution-based methods [26]. Anchor-based methods are based on the comparison of patient-rated outcomes to an anchor, that is, the patient's global impression of improvement [27]. Distribution-based methods use standard error measurement (SEM), standard deviations, and effect size [28, 29]. The use of MCIDs facilitates our ability to determine even small improvements in pain scores for this feasibility study. Thus, this study was designed to explore the acceptance of APA to reduce CLBP, assess subject adherence, assess safety/tolerability (i.e., somatic symptoms), and estimate minimum clinically important differences for pain intensity change.

## 2. Methods

This study employed a repeated-measures observational design, and subjects received a 7-day auricular point acupressure (APA) research protocol for the assessment and management of CLBP. Data (pain severity, pain interference, and medication use) were collected at baseline, daily for 7 days (total 8 time points). 

### 2.1. Subjects and Setting

Subjects with CLBP independently approached the PI with requests to participate in the study when Dr. Yeh was recruiting subjects with cancer-related pain from the UPMC (University of Pittsburgh, Medical Center) Cancer Center follow-up clinic in McKeesport (a suburb of Pittsburgh, 10 miles from the main campus). Those subjects were friends and family of participants in the cancer pain study. Thus, the IRB was revised to expand the study to recruit subjects with CLBP. Subjects were eligible for this study if they were 18 years of age or above, had nonspecific low back pain for more than 6 months, pain intensity ≥ 3 on a 10 point numerical pain scale at the time of recruitment, had no surgery in previous 3 months, had not received any acupuncture or acupressure treatments in the previous three months, and were able to read and write English. 

### 2.2. Measures

We used the Brief Pain Inventory (BPI) [30] to assess the severity and impact of pain on daily functions in the previous 24 hours. Included were front and back body diagrams, pain intensity rating (4 items: worst pain, least pain, average pain, and current) and pain interferences (7 items) using 0–10 scales, as well as pain medication used and the percentage of pain relief by pain medication. Two singular items of worst pain, average pain, a composite of the 4 items of pain severity and pain interference were used as the outcome variables. Higher scores indicated that patients had higher pain intensity. For data analysis, the score for each outcome variable was standardized so that each outcome variable had potential score of 0–10. 

Analgesic use was monitored on the subject's diary and the BPI. The Medication Quantification Score Version III (MQS) [31] was used to compute a single numeric value for a subject's pain medication profile. This score was based on the subject's use of drug by class, dose, and detriment (risk). The decreased MSQ III score was associated with improved outcome (less pain intensity) [31]. The classification of the drug class followed the suggestion of World Health Organization (WHO) level 1–3 analgesic drugs, coanalgesic drugs (tricyclic antidepressants, antiepileptics), and other drugs such as benzodiazepines or muscle relaxants.

The Perceived Therapeutic Efficacy Scale was administered to examine subjects' expectations regarding the benefit of acupressure. This scale was adapted from the Perceived Treatment Efficacy Assessment in Rheumatoid Arthritis scale [32]. 

The subjects' satisfaction with the treatment and the extent to which they perceived the treatment to be a burden was assessed with a modified version of the Satisfaction Questionnaire used by a previous study [33]. Subjects were interviewed by phone at the end of the study regarding their subjective experiences during the APA intervention and were called daily to assess their pain intensity, stimulation times (frequency), and stimulation duration for acupoints. One item of the patient-rated 5-category ordinal assessment measures the patient's general pain improvement and was scored as (1) much better, (2) better, (3) about the same, (4) worse, and (5) much worse. 

### 2.3. Auricular Point Acupressure Treatment Protocol

**Figure 1:**
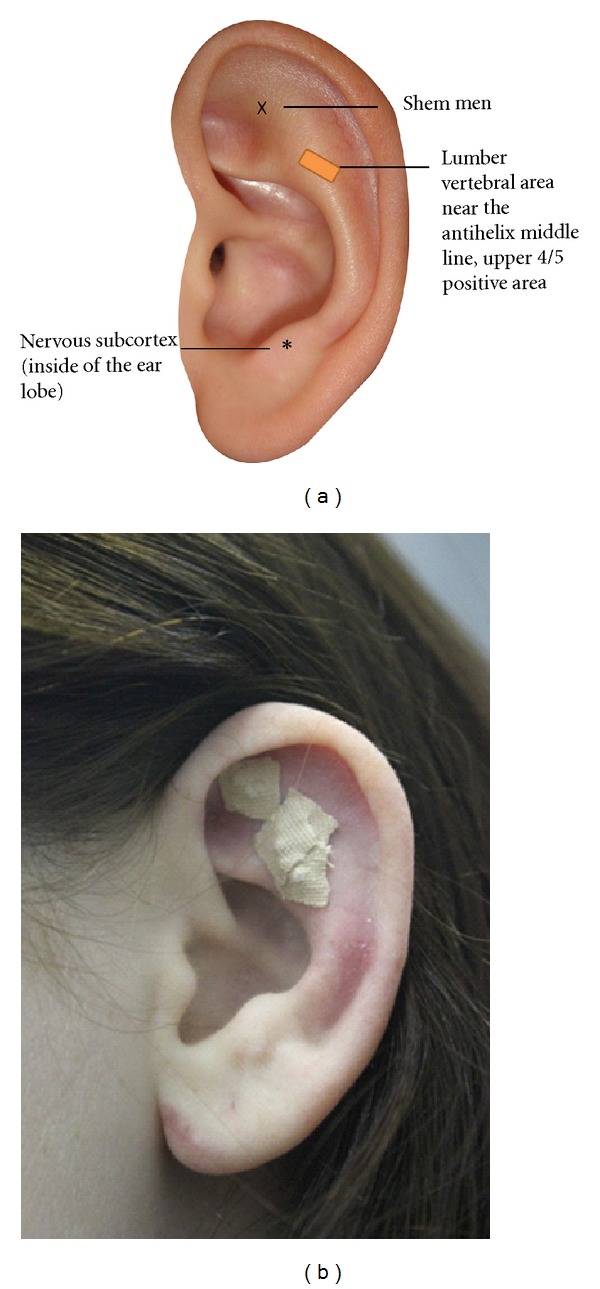
Auricular acupoints for low back pain treatment.

The auricular points selected for pain treatment included two commonly used acupoints (Shenmen and nervous subcortex) as well as the acupoints corresponding to where patients had pain, including the lumber vertebral area near the antihelix middle line and the upper 4/5 positive area (front and back ear area) (see Figure 1). The Shenmen point has been recognized as having wide application for pain [34], and the nervous subcortex point is related to vasodilation in the holistic nature of the therapy [11]. The number of acupoints and the locations on the ears for each patient may be different because each patient may have had different locations of pain and the pain is projected onto the ear according to somatic topography. The selection of corresponding points was made according to Dr. Huang's ear reflex theory [35, 36] and personal communication with Dr. Huang (Nov 27, 2010). Dr. Huang, who received the Recognition of Mastery and Lifetime Achievement Award at the World International Symposium on Auricular Therapy & Auricular Medicine (2002), is regarded as the “Mother of Auricular Therapy” in China and is the President of Auricular Medicine International Research & Training Center, Florida [11]. Dr. Yeh, the first author of this study, studies with Dr. Huang and is a certified auricular therapist. 

**Figure 2:**
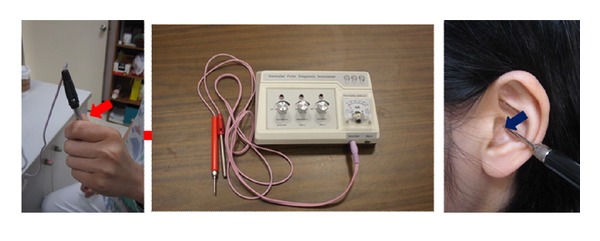
Acupoint finder.

**Figure 3:**
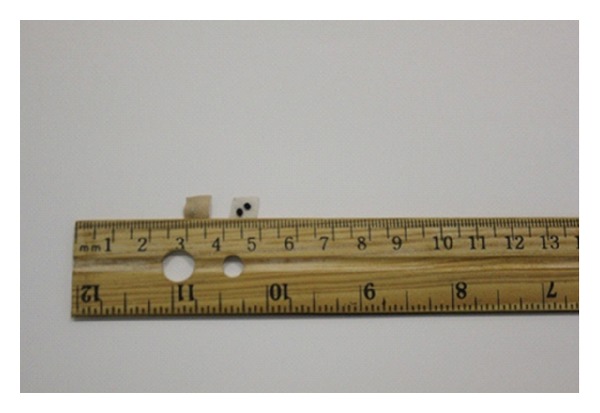
Vaccaria seeds with tan colored tape.

Acupoints on each subject were identified using an electronic acupoint locator. The acupoint locator (Figure 2) was connected to two probes: one was held by the subject and the other was used by the PI to locate the acupoints. The acupoint was identified when the locator made a sound indicating the corresponding location on the body. Vaccaria seeds (Figure 3) were carefully taped onto each selected auricular point. We demonstrated the pressing technique to subjects before asking them to do a reciprocal demonstration. Moderate stimulation was used for the therapy. Subjects were told that they should press the seed-tapes with increasing pressure until they felt either slight discomfort or a tingling sensation. Subjects were asked to press each of their taped acupoints at least 3 times a day for at least 3 minutes for each of the seven days of the study even if they did not have symptoms. They were also instructed to press the seed-tapes whenever they experienced pain. Tapes were kept on auricular points for 7 days, and patients were asked to remove them on day eight. 

### 2.4. Procedure

After the consent form was signed by the subjects, subjects received the treatment by Dr. Yeh. A data collector, who was not Dr. Yeh, called the subjects daily to remind the subjects to perform APA intervention (i.e., frequency and duration) at home, document any side effects of APA, and collect the outcome measures (BPI short form and medication use) for the past 24 hours. All of the data were entered by this trained data collector. 

## 3. Data Analysis 

Descriptive statistics were used to examine the demographic characteristics of all (*n* = 64) participants. We used the generalized additive model [37] to accomplish a longitudinal data analysis for evaluating the APA intervention on the pain outcomes (severity and interference) before and daily for 7 days of assessment. Four continuous measurements (worst pain, average pain, overall pain severity, and pain interference) were investigated.

In order to estimate minimum clinically important differences (MCIDs) for the BPI average pain, worst pain, and severity score (the mean of the BPI pain scale values: right now, average, least, and worst), the treatment satisfaction measure of “subject's overall impression of pain improvement” was used as an anchor. Pain improvement is a patient-rated 5-category ordinal assessment that measures the patient's general level of improvement and is coded as follows: (1) (much better), (2) (better), (3) (about the same), (4) (worse), and (5) (much worse). End point scores of (1) (much better) and (2) (better) were used to identify patients who had an overall clinically important difference, also called “minimal clinically relevant improved,” whereas the “stable” group was the subjects who rated their pain improvement as (3) (about the same). The MCID was calculated as the difference in the unadjusted mean change in the BPI scores between the “stable” group and “improved” group. In final analysis of MCIDs, only subjects who had complete data were used (*n* = 36 for Improved group and = 22 for Stable group, 6 subjects were not included due to missing data). MCIDs were also expressed as a percentage reduction from the mean baseline scores for the stable and improved groups of each measure. All data analysis was performed using SAS software, version 9.2.3 (SAS Institute Inc., Cary, NC).

## 4. Results

### 4.1. Demographics

Table 1 presents the demographic characteristics of the 64 subjects who completed the study (24 males and 40 females). The mean age in years was 63.70 (SD = 14.00). Most of them (>90%) were cohabitating and live at home. A total of 74 subjects approached the study coordinator and requested participation in our APA study. During the data collection, 3 decided to drop out due to no effects of APA, 2 were hospitalized during data collection, 2 felt too much pain on their ear, 2 had allergic reactions to the tape, and 1 was out of town after participation in the study. The retention rate was 87% (64/74). Patients who stayed in the study were all able to adhere to the APA protocol at home (at least 3 times/day, 3 minutes/time).

### 4.2. Pain Intensity and Pain Interference Change

Table 2 shows pain intensity and interference change from baseline to day 7 (completion of the APA treatment). For the subjects as a whole, the BPI worst pain score had decreased from baseline (mean = 7.61, SD = 1.63) to day 1 (the first day of the APA treatment) (mean 4.60, SD = 2.81, a reduction of 40%) and maintained about the same degree of worst pain score at day 1 over the course of APA (mean ranged from 3.54 to 4.59). The percentage decrease at day 6 reached 54% and was the only day with over 50% percentage decrease while receiving auricular acupressure. Averaged pain and overall pain severity had similar change patterns of worst pain and had over 55% pain reduction by the end of the 7-day APA treatment. The mean score of pain interference was 4.63 (SD = .31) and had reached the highest decrease at day 6 (mean = 1.6, a reduction of 64%). 

### 4.3. Minimum Clinical Important Differences (MCIDs)

**Figure 4:**
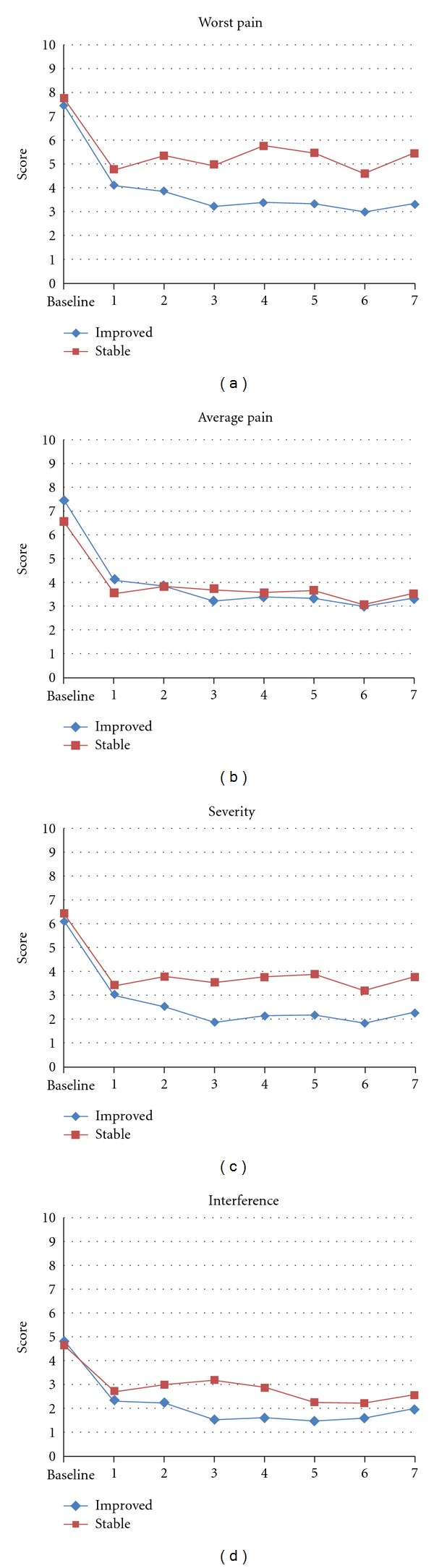
Change patterns of worst pain, average pain, pain severity, and pain interference from baseline to day 7 between subjects who were self-rated “Improved” and “Stable” groups.

According to the “Pain Improvement” questionnaire, 36 subjects rated themselves as “improved,” whereas 22 subjects rated themselves as “stable.” Table 3 lists the MCIDS for worst pain, average pain, and pain intensity at baseline and by the end of APA treatment (end point) for both “Improved” and “Stable” groups. The change patterns for pain score change and pain interferences for 8 data point times are shown in Figure 4. The MCIDs using the Patient Satisfaction score as the anchor were an improvement of −1.86 points for BPI worst pain, −0.84 for BPI average pain, −1.16 for BPI severity, and −0.70 for BPI interference. The MCIDs expressed as percentage of improvement from baseline were 24.9% for the BPI worst pain score, 12.3% for BPI average pain score, 19.0% for the BPI pain severity, and 14.5% for BPI pain interference. 

### 4.4. Patient Satisfaction

Fifty-five subjects (86%) reported fewer episodes of pain and improved pain (Table 4). Sixty-nine percent of patients took less pain medication than before treatment; 62% subjects felt “much better” after the APA treatment. The mean improvement percentage of pain after APA was 57.02 (SD = 25.23). Only one patient reported feeling worse. Only 8% subjects were not satisfied with the APA.

## 5. Discussion

Our study showed that subjects with CLBP reported a 46% reduction in BPI worst pain, 54% reduction in BPI average pain, 56% reduction in BPI pain severity, and 55% reduction in BPI pain interference after 7 days of APA. By the end of the study, 62.5% of subjects also reported less pain medication use during the APA treatment. The retention rate for this study was 87% for the 7-day intervention of APA. The MCIDs for BPI worst pain, BPI average pain, BPI pain severity, and BPI pain inferences ranged from  .70 to 1.86 points. The percentage improvement of MCIDs from baseline was 14.5–24.9%. 

Before interpretation of the study findings, several study limitations must be acknowledged. First, the subjects in this study approached the study coordinators to request participation in the study. Thus the high retention rates cannot be extrapolated to the general population, who may not be interested in complementary and alternative medicine (CAM). Second, our study did not have a placebo-control group, so we are not able to differentiate the true effects of APA from the possible psychological effects of the patients' expectation of APA treatment and daily phone calls by the data collectors. We did not investigate the causes of CLBP, and the follow-up period was short in this study, limiting the conclusions that can be drawn. Another limitation of this feasibility study was that the subjects were derived from a convenience sample of individuals who accompanied cancer subjects to a cancer study center study site for APA, and these individuals requested that we let them try APA for their CLBP. We did not collect data from CLBP center and thus did not have access to an expert in CLBP as part of our team. We were very surprised at the number of people who came forward wanting to try APA for CLBP, and we did not know if it would help them. In a future clinical trial on subjects with CLBP, we would include at least one CLBP expert on our team. In addition, a CLBP-related dysfunction measure, namely, Roland-Morris Disability Questionnaire (RMDQ) [38, 39] would be used to assess the impact of daily functioning (back-related). 

During the APA treatment, two patients dropped out of the study due to their allergy to the tapes. The tape we used for APA treatment contained latex (provided by Auricular Medicine International Research & Training Center, Florida, US). A latex-free, hypoallergenic, and gentle tape will be used in any future study to reduce skin irritation. Approximately 2/3 patients reported pain on their ears when they pressed the tapes in the first couple of days and the pain sensation on their ear decreased gradually. This pain sensation on the ear is much higher than the previous study using auricular acupuncture, in which the needle stayed in situ for 48 hours (14%) [20]. Most subjects in our study indicated that they were comfortable with the therapy after a couple of days and expressed their willingness to endure the ear pain as long as their CLBP could be relieved, although two subjects (3%) decided to drop out due to the sharp pain in their ear when they pressed the tapes. Thus, it is important to advise the subjects about the potential side effect of pain when they receive APA treatment. During the study period, a trained data collector called the subjects daily to query their APA practice and their pain intensity. All of the subjects followed the APA instructions. Over 90% subjects also indicated that they followed the frequency, duration, and time for the APA practice at home.

The pain intensity change (BPI worst pain, average pain, and pain severity) by the end of the 7-day APA treatments is lower in over 47% in subjects when compared with baseline, which is higher than that reported in auricular acupuncture studies of CLBP [14, 18, 20]. In a combination auricular acupuncture and exercise 12-week intervention program, subjects with CLBP showed mean improvement of pain intensity (−0.93 point at by the end of intervention and −2.08 at 6 month follow up) [20]. The comparison of 1-week of pain intensity change was not available since data was not shown for this study. The greater improvement of our study after 1-week APA shows the potential benefit to reduce CLBP. Further studies are needed to validate the analgesic effects of APA treatment for longer duration following the treatment.

The MCIDs (mean ranged from 0.74 from 1.86 and 12–25% change) calculated in this study were lower than other previous studies, which had approximately a 2-point improvement (35–55% improvement) [27, 40]. These estimates are greater than our study findings. These differences may be due to the different patients studied, pain types, and study design. In our study, we assessed only a 1-week APA treatment, without control/placebo groups. We used patients' rated improvement on a scale of a 5-category assessment and previous studies used scale of 7-category ordinal assessment as the anchor for the BPI. Therefore, our study had a lower response profile. 

APA is an extremely affordable therapy to practice and research. One tape with seeds costs about $0.12 and every APA treatment may cost from $1.2 to $2.4, depending on how many tapes were used. The most significant advantage of APA is that once the tapes are placed by a trained practitioner, the patients can press the acupoints at home by themselves. In contrast, subjects who receive acupuncture treatment need to visit the therapist's office to receive the treatment administered by the registered acupuncturist. The number and frequency of acupuncture treatments vary, but a minimum of 12 sessions of acupuncture is suggested for CLBP to get the most benefits (2 sessions per week for 4 weeks and then 1 session per week for 4 weeks) [10]. The cost of acupuncture treatment varies and may range from $65 to $125 per session [41]. Medicare and Medicaid do not cover acupuncture; however, the proportion of third-party plans providing coverage is increasing [42]. In addition, acupuncture treatment for CLBP is not necessarily lower in cost. Studies have shown no cost savings in back care services after 1 year among groups receiving acupuncture compared with patients who received massage or self-care [43] or have shown a modest increase in overall treatment costs [44, 45] when in adjunct to usual care. Although auricular acupuncture, which can leave the needle in situ for up to 1 month, may solve the problems of frequent therapist office visits [18, 46], it must be administered by a licensed therapist. If APA can achieve treatment effects comparable to acupuncture, the use of APA will receive far more application in clinical settings. The tapes may be able to stay on subject's ear longer—up to two to three weeks—if the subjects do not have allergic reactions to the tape. APA can be administered by a broader range of health care professionals because APA does not require insertion of needles. Health care professionals such as nurses, physical therapists, and psychologists can be taught through continuing education, and then be able to incorporate APA into clinical practice. 

Consistent with previous studies [14], subjects in the current study decreased the use of pain medication during the APA treatment. All of the subjects indicated their desire to reduce pain medication when they enrolled in the study. In addition to the cost saving in medication use, the reduction of pain medication used (such as nonsteroidal anti-inflammatory drugs and opioids) can also decrease the risk of potential side effects (i.e., gastrointestinal bleeding, nausea, vomiting, constipation, and dizziness [47, 48]). 

In this study, we tested only a 7-day APA treatment. The effects of APA reached the maximal pain reduction on Day 4 and decreased after Day 5. Dr. Huang has suggested that a course of APA treatment should include a cycle of 4 treatments (5-day continuous treatment with 2 days off and repeated for 4 weeks) [11]. This treatment duration is similar to suggested acupuncture treatment [10]. Further studies are needed to determine the optimal duration of treatment to achieve sustained therapeutic effects. 

In order to examine the true effects of APA, a large-scale randomized clinical trial would be required, including a sham group, tracking of long-term effects, and examination of a longer treatment duration (i.e., 4-week or 6-week [18]) to obtain the maximum effects. In addition, understanding the underlying biological mechanisms associated with APA may explain how the neuroendocrine, immunologic, and other physiological processes are related to auricular acupressure analgesia.

## Figures and Tables

**Table 1:** Demographic characteristics of the subjects.

	*N* (%)
Age mean (standard deviation) (range)	63.70 (14.00) (22–89)
Gender	
Male	24 (38)
Female	40 (62)
Living status	
Cohabitating/married	55 (86)
Other (live alone)	9 (14)
Marital status	
Married/partnered	49 (77)
Divorced/separated/other	15 (23)
Education	
Primary	3 (5)
Secondary	30 (47)
College and above	31 (48)
Ethnicity	
White	58 (91)
Black	5 (8)
Asian	1 (1)
Medication use	
Yes	47 (63)
No	17 (27)
Mean pain intensity score at baseline	Mean (standard deviation)
Worst pain	7.62 (1.62)
Average pain	6.63 (1.93)
Pain severity	6.27 (1.84)
Pain interference	4.88 (2.75)

**Table 2:** The trend of mean pain score and interference from baseline to day 7 and the difference of mean pain score between each treatment day and baseline.

	Worst pain			Average pain			Severity			Interference		
	Mean	(SD)	Change (%)	Mean	(SD)	Change (%)	Mean	(SD)	Change (%)	Mean
D0	7.61	(1.63)		6.33	(1.94)		6.29	(1.85)		4.88	(2.77)	
D1	4.60	(2.81)		3.42	(2.42)		3.41	(2.39)		2.70	(2.78)	
D2	4.59	(2.87)		3.24	(2.70)		3.25	(2.44)		2.66	(2.86)	
D3	4.06	(2.99)		2.77	(2.66)		2.79	(2.42)		2.27	(2.64)	
D4	4.42	(2.87)		2.92	(2.48)		3.01	(2.38)		2.23	(2.58)	
D5	4.14	(2.85)		2.86	(2.48)		2.94	(2.35)		1.89	(2.52)	
D6	3.54	(2.71)		2.30	(2.16)		2.49	(2.15)		1.89	(2.48)	
D7	4.13	(3.15)		2.89	(2.72)		2.96	(2.57)		2.31	(2.65)	
D1 versus D0	−3.01	(2.27)	−39.57%	−2.91	(2.18)	−46.01%	−2.88	(2.12)	−45.79%	−2.18	(2.18)	−44.61%
D2 versus D0	−3.03	(2.29)	−39.75%	−3.09	(2.33)	−48.78%	−3.04	(2.14)	−48.38%	−2.22	(2.22)	−45.52%
D3 versus D0	−3.56	(2.33)	−46.71%	−3.55	(2.28)	−56.17%	−3.50	(2.12)	−55.66%	−2.61	(2.61)	−53.41%
D4 versus D0	−3.19	(2.25)	−41.95%	−3.41	(2.19)	−53.86%	−3.28	(2.09)	−52.13%	−2.65	(2.65)	−54.24%
D5 versus D0	−3.47	(2.23)	−45.57%	−3.47	(2.19)	−54.85%	−3.34	(2.08)	−53.18%	−2.99	(2.99)	−61.25%
D6 versus D0	−4.07	(2.16)	−53.49%	−4.03	(2.04)	−63.66%	−3.80	(1.98)	−60.48%	−2.99	(2.99)	−61.26%
D7 versus D0	−3.48	(2.42)	−45.72%	−3.44	(2.32)	−54.38%	−3.33	(2.20)	−52.89%	−2.57	(2.57)	−52.69%

D: day, SD: standard deviation.

**Table 3:** Estimation of mean changes in BPI average pain and BPI severity scores^∗^.

		Subject numbers	Baseline	End point	Mean change	
Pain Score	Anchor status^†^	Mean (SD)	Mean (SD)	Mean (SD)	Mean (SD)	MCIDs^‡^
Worst pain	Improved	36	7.47 (1.70)	3.32 (2.96)	−4.15 (2.37)	−1.86 (45.72)
	Stable	22	7.76 (1.67)	5.47 (3.12)	−2.29 (2.42)	
Average pain	Improved	36	6.03 (1.84)	2.25 (2.52)	−3.78 (2.18)	−0.74 (54.38)
	Stable	22	6.57 (2.04)	3.53 (2.53)	−3.04 (2.27)	
Severity	Improved	36	6.09 (1.94)	2.27 (2.18)	−3.83 (2.05)	−1.16 (52.88)
	Stable	22	6.45 (1.86)	3.78 (2.59)	−2.67 (2.22)	
Interference	Improved	36	4.82 (2.69)	2.02 (2.50)	−2.80 (2.60)	−0.70 (52.69)
	Stable	22	4.65 (2.94)	2.55 (2.84)	−2.10 (2.90)	

MCIDs: minimum clinically important differences.

^†^Grouped according to responses from treatment satisfaction questionnaire.

^‡^Expressed as score reduction (Improved, Stable) and percent reduction from baseline.

**Table 4:** Satisfaction of auricular point acupressure treatment for pain.

	*N* (%)
Fewer episodes pain	
Yes	55 (86)
No	9 (14)
Pain improved	
Yes	55 (86)
No	9 (14)
Take less medication than before treatment	
Yes	44 (69)
No	20 (31)
Overall feeling	
Much better	40 (62)
Better	24 (15)
About the same	13 (8)
Worse	1 (1)
How much better mean (SD)	57.02 (25.23)
Satisfaction about the progress	
Completely	45 (71)
Somewhat	14 (21)
Not satisfied	5 (8)
